# Global Warming: Predicting OPEC Carbon Dioxide Emissions from Petroleum Consumption Using Neural Network and Hybrid Cuckoo Search Algorithm

**DOI:** 10.1371/journal.pone.0136140

**Published:** 2015-08-25

**Authors:** Haruna Chiroma, Sameem Abdul-kareem, Abdullah Khan, Nazri Mohd. Nawi, Abdulsalam Ya’u Gital, Liyana Shuib, Adamu I. Abubakar, Muhammad Zubair Rahman, Tutut Herawan

**Affiliations:** 1 Faculty of Computer Science and IT, University of Malaya, Kuala Lumpur, Malaysia; 2 Software and multimedia center faculty of science and computer technology, University Tun Hussein Onn, Johor Bahru, Malaysia; 3 Faculty of Computing, University Technology Malaysia, Johor Bahru, Malaysia; 4 Faculty of Information and Communication Technology, International Islamic University Malaysia, Kuala Lumpur, Malaysia; 5 School of Science, Department of Computer Science, Federal College of Education (Technical), Gombe, Nigeria; University of Vermont, UNITED STATES

## Abstract

**Background:**

Global warming is attracting attention from policy makers due to its impacts such as floods, extreme weather, increases in temperature by 0.7°C, heat waves, storms, etc. These disasters result in loss of human life and billions of dollars in property. Global warming is believed to be caused by the emissions of greenhouse gases due to human activities including the emissions of carbon dioxide (CO_2_) from petroleum consumption. Limitations of the previous methods of predicting CO_2_ emissions and lack of work on the prediction of the Organization of the Petroleum Exporting Countries (OPEC) CO_2_ emissions from petroleum consumption have motivated this research.

**Methods/Findings:**

The OPEC CO_2_ emissions data were collected from the Energy Information Administration. Artificial Neural Network (ANN) adaptability and performance motivated its choice for this study. To improve effectiveness of the ANN, the cuckoo search algorithm was hybridised with accelerated particle swarm optimisation for training the ANN to build a model for the prediction of OPEC CO_2_ emissions. The proposed model predicts OPEC CO_2_ emissions for 3, 6, 9, 12 and 16 years with an improved accuracy and speed over the state-of-the-art methods.

**Conclusion:**

An accurate prediction of OPEC CO_2_ emissions can serve as a reference point for propagating the reorganisation of economic development in OPEC member countries with the view of reducing CO_2_ emissions to Kyoto benchmarks—hence, reducing global warming. The policy implications are discussed in the paper.

## Introduction

Global warming and the effects of greenhouse gases are considered among the important issues in the fields of science and politics [1–2]. This has triggered increasing concern about the contributions of carbon dioxide (CO_2_) to global warming [3]. The Intergovernmental Panel on Climate Change pointed out that more than 90% of global warming is probably caused by the emission of greenhouse gases due to human activities. The negative impacts of Global warming across the globe are as follows: The observed increase in temperature to date is about 0.7°C, which has started affecting health in several societies across the world. Extreme weather is increasing, especially heat waves, floods, and storms, which results in an increasing loss of human life and injuries due to natural disasters caused by climate change. The determinants of health, such as quality and quantity of foods, water resources, and ecological disease control vectors, are also affected [4]. In addition, community structures are expected to be influenced by Global warming [5].

Energy consumption is viewed as the major source of greenhouse emissions [6]. Energy consumption from 1970–2010 for the Organization of the Petroleum Exporting Countries (OPEC) has increased by 685%, while the emissions of CO_2_ increased by 440% as a result of burning fossil fuels within the same period. Therefore, energy consumption and CO_2_ emissions of the OPEC countries have drastically increased [7]. The burning of fossil fuels, has increased the global temperature caused by CO_2_ emissions [8]. The OPEC countries contributed 7% of the world CO_2_ emissions in 2010. This is considered to be significant for the use of energy in the future and for the potential of greenhouse emissions from the OPEC countries. Global warming is one of the critical issues currently facing the world. The trend of oil consumption and CO_2_ emissions of OPEC countries has grave implications by contributing to global warming [7]. The world is affected by the dangers of global warming, and the major contributor to global warming among the greenhouse gases is CO_2_ emissions [6]. As a result, the attention of policy makers and governments throughout the world has been focused on creating a framework based on energy efficiency simulation capable of conserving energy, thereby reducing the consumption of energy and the emission of greenhouse gases [7]. Reducing greenhouse gases emitted as a result of energy consumption, reduces the effects of global warming [9–10].

The emission of CO_2_ requires an accurate prediction for close monitoring and control [11]. Predicting CO_2_ is significant for the adaptation of climate change policies as well as for offering a reference point for using alternative energy sources [12–14] with the view to reduce CO_2_ emissions [15].

The creation of preventive measures for reducing CO_2_ emissions has motivated attempts in the literature to apply computational intelligent algorithms due to their superiority over formal logic, mathematical programming [16], and statistical methods [17] for predicting the emissions of CO_2_. Despite the limitations of the these traditional methods, Meng *et al*. [18] used a non-homogeneous exponential equation and a linear equation to build a model for the prediction of energy related CO_2_ emissions in China. To avoid the limitations of the traditional methods, Chen and Wang [19] applied a hybrid of fuzzy regression and backpropagation neural network (BPNN) (FRBPNN) to forecast the global concentration of CO_2_. It was found to improve the accuracy of CO_2_ forecasting. Chen [11] used a collaborative fuzzy neural network to improve the forecast accuracy of the FRBPNN. Results indicated that the collaborative fuzzy neural network outperforms the FRBPNN and statistical methods in the forecasting of global CO_2_. Bao and Hui [20] applied the Grey model to build a model for the forecasting of CO_2_ emissions in Shijiazhuang, China. The model was used to project the CO_2_ emissions of Shijiazhuang from 2010 to 2020. In another study, the CO_2_ emissions related to energy in developing countries were forecasted using the Grey model [21]. The Grey model is not effective with a large sample of data; it requires small samples of observations to be robust [22], lacks fitting ability and has a deficiency in nonlinear modeling [23]. This motivated Tan and Zhang [23] to use GA to improve fitting ability of the Grey model and combined the genetic algorithm (GA) fitted Grey into BPNN for improving its nonlinear approximation ability. The model was used to predict energy load with improved performance.

However, the BPNN is a gradient based algorithm that has the possibility of being stuck in local minima, slow convergence, highly dependent on parameter settings, and generates complex error surfaces with a multiple local minimum [24–25]. Fuzzy systems lack the capability of learning input data; human language is used to represent the input and output of the systems. Thus, incomplete or wrong rules cannot be handled well by fuzzy systems. Tuning of the systems is not a direct task [26]. The GA abolished previous knowledge of the problem if the population changes [27], and requires many parameter settings that undermine its robustness [28].

Studies on the prediction of OPEC CO_2_ emission from petroleum consumptions are scarce in the literature, despite the increasing consumption of petroleum and emissions of CO_2_ by the OPEC countries. Limitations of the previous studies and lack of work on the prediction of OPEC CO_2_ emission from petroleum consumptions motivated the present research.

To circumvent the limitations of the gradient decent algorithms, several biologically inspired global algorithms were proposed such as GA, particle swarm optimisation (PSO), artificial bee colony (ABC), etc., and recently cuckoo search algorithm for training the ANN. However, the cuckoo search algorithm (CS) was found to be more effective than the GA, PSO, and ABC [29]. In this paper, we proposed to hybridise the CS and Accelerated PSO (APSO) for training ANN (HCSNN) to build a model for the prediction of OPEC CO_2_ emissions. The HCSNN can improve the prediction accuracy and convergence speed of the ANN more than the GA, ABC, CS, and APSO as shown in the preliminary experiments [30].

In our approach, the hybrid CS communication capability of the cuckoo births has been improved by introducing APSO to search for a better location in which the optimal nest can share information with the cuckoo unlike in the previous studies. In the literature, Valian *et al*. [31] modified the CS by using variable probability of worse nests and step size when generating new solutions instead of the constant probability of worse nests and step size. Abubakar *et al*. [32] adopted Walton *et al*. [33] modified CS by adding exchange information between the eggs to the model and the crossover. Also, the distance to the location of a new egg was computed using an inverse golden ratio. Abubakar *et al*. [32] used the Walton *et al*. [33] modified CS to train a Functional Link ANN to build a model for the prediction of temperature and relative humidity in Malaysia. Abubakar *et al*. [34] further applied the model proposed in [32] for the prediction of climate change via temperature and ozone.

## The Proposed Methods

### Cuckoo Search Algorithm

The Cuckoo search algorithm is a new optimisation algorithm [35] developed by Yang and Deb [36], currently attracting attention from the research community. Attention is expected to continue into the future [28]. The CS is a global search algorithm for searching a global optimum solution. In CS, the fitness can be proportional to the objective function value without difficulties. Getting an optimised solution of a complicated problem using CS does not require a comprehensive search. Cuckoos are fascinating birds due to their aggressive strategies in reproduction. The 3 types of the brood parasitism strategy are as follows: (1) Intraspecific brood parasitism; (2) cooperative breeding; (3) nest takeover. Engaging in conflict directly between the host birds and cuckoos is possible. The host birds either abandon the nest or throw the alien eggs out of the nest to produce new eggs. In lévy flight distribution, animals and birds search for food in random or quasi-random, thus following a random walk, since the subsequent action relies on the present position and transition probability of the next state [37]. This behaviour has been applied in the CS optimisation, which has shown a better performance than other distribution-based random walk in exploring large scale search space. The lévy flight distribution is expressed as shown in Eq (1) based on Fourier transform (*I*) Yang [38]:

$$I(s)=l^{\left[-\alpha {\left|s\right|}^{\lambda }\right]}\;0<\lambda \leq 2$$ (1)

Where *α* is the scaling parameter and *s* is the step length. Only special cases of parameters have inverse transform with explicit analytical formulae. Eq (1) can be changed to Eq (2) if the λ = 2.

$$I(s)=l^{\left[-\lambda s^{2}\right]}$$ (2)

The inverse integral of the transform of Eq (2) produces the Gaussian distribution and the inverse integral is expressed in Eq (3):

$$M(s)=\frac{1}{\pi }\int_{0}^{\infty }\;\text{cos}(ns)l^{\left[-\lambda {\left|n\right|}^{\mu }\right]}dn$$ (3)

Where *M* is the cost function and *μ* is the location parameter, when *s* → ∞ Eq (3) becomes:

$$M(s)=\frac{\lambda \mu \Gamma (\mu )\text{sin}(\pi \mu /2)}{\pi {\left|s\right|}^{1+\mu }}$$ (4)

$$\Gamma (y)=\int_{0}^{\infty }f^{y-1}l^{-f}df$$ (5)

Where the gamma function is represented by Γ(*y*) and *y* = *n*, we have Γ(*n*) = (*n*−1)!. The 3 major ideas of the CS proposed by Yang and Deb [36] for rules as an optimisation algorithm for the CS are: (1) Each of the cuckoo lays one egg at a time and puts it in a randomly chosen nest; (2) the nests with the optimum quality eggs will move to the next generation; (3) the available nest host is fixed and the egg laid by a cuckoo is discovered by the host bird with the probability of worse nests to be abandoned (*P*_*a*_) *p*_*a*_ ∈ [0,1]. The fitness function is selected as the objective function itself for maximum or minimum problems. In the generation of a new solution, $x_{i}^{\left(t+1\right)}$for cuckoo *i*, a levy flight is performed as expressed in Eq (6):

$$x^{\left(t+1\right)}=x_{i}^{t}+\alpha_{1}⊕levy(\lambda )$$ (6)

**Fig 1:**
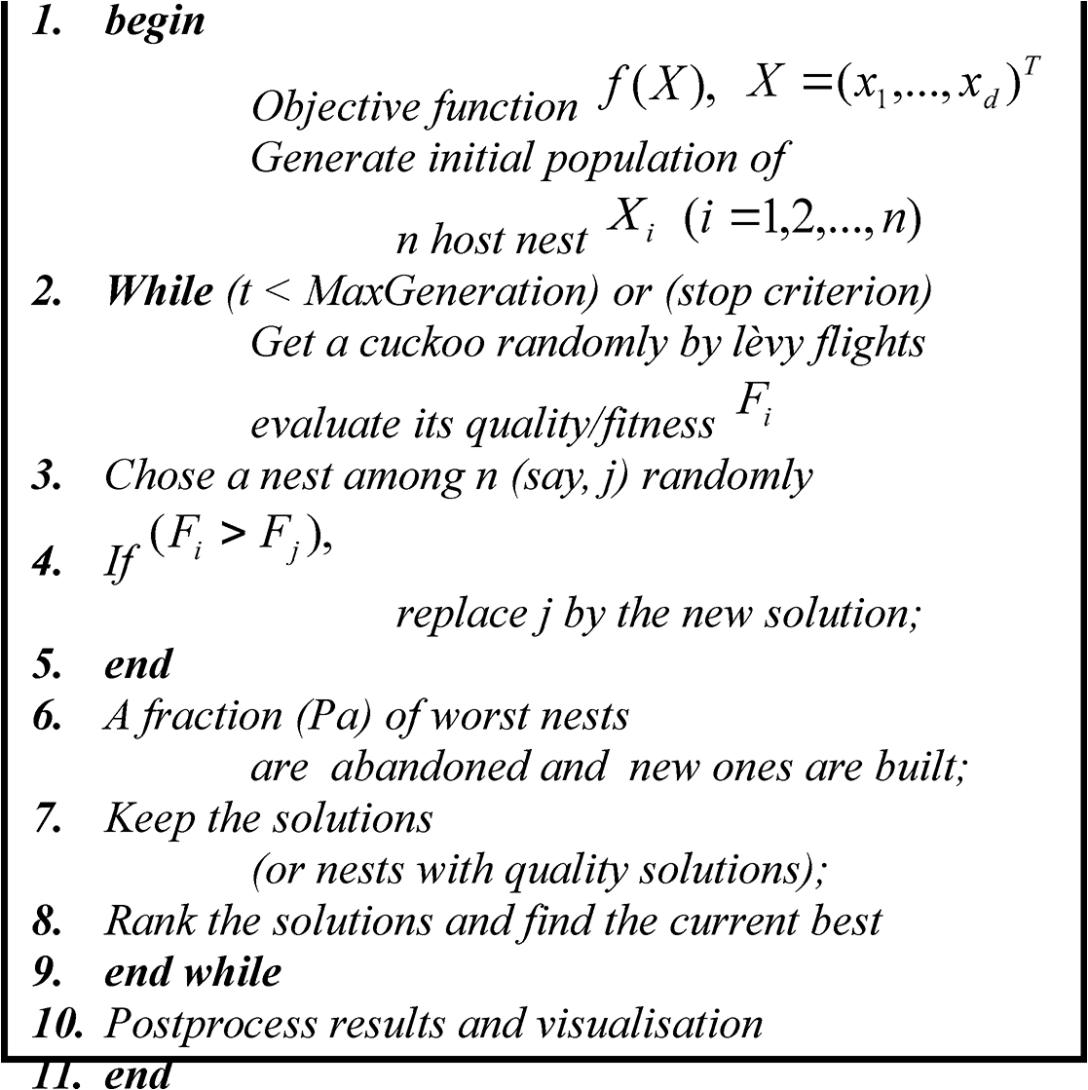
Pseudo-code of CS.

Where α_1_ is the lévy flight step size multiplication processes with an entry wise multiplication process. However, levy flight provides a random walk, whereas their random step lengths are drawn from the levy flight distribution for large steps. The CS initialised the population (*n*) for the nest, and randomly selected the best nest via levy flight. Thus, the cuckoo birds are always looking for a better place in order to reduce the chance of their eggs being discarded. The CS requires the setting of parameters for execution such as *n*, etc. However, the most critical parameters required to obtain the optimal solution from CS are *P*_*a*_ and α_1_ [39]. The pseudo-code for the CS is shown in Fig 1.

### Accelerated Particle Swarm Optimisation

#### Particle Swarm Optimisation

The choreography behaviour of birds and insects motivated Kenneth and Eberhart [40] to propose PSO. A number of individuals in PSO refined their knowledge of the given search space. Each and every individual in a PSO has a particle that refers to position and velocity. In PSO two pieces of information are responsible for adjusting the particle trajectory: The best location stays at the present point and global best location is reached by the entire swarm. The PSO uses evaluation function to assign a fitness value like other optimisation techniques.

**Fig 2:**
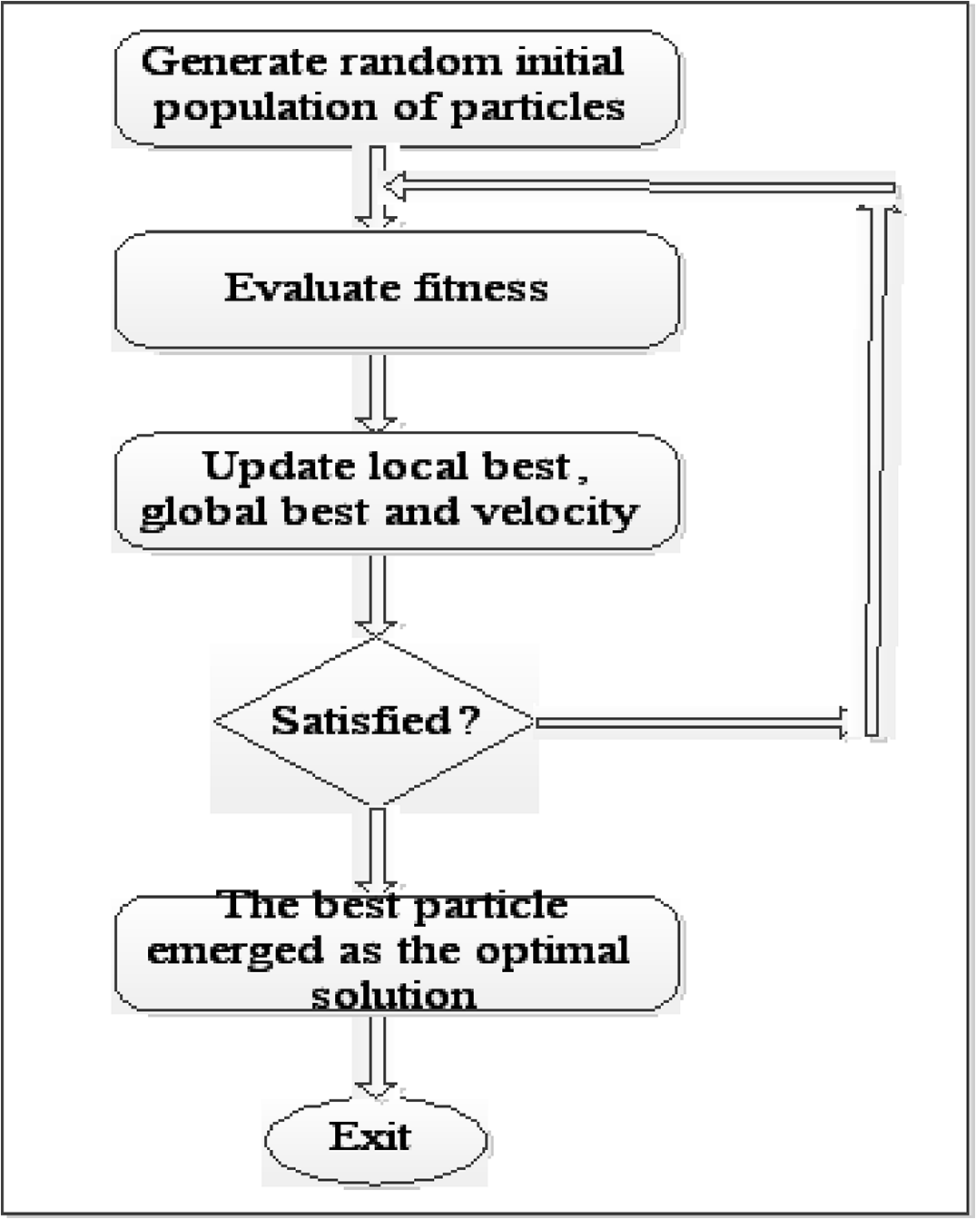
The basic stages of the original PSO.

Global best is the highest fitness value reached by a swarm, while local best is the highest fitness value that an individual particle has attained. Global and local best are remembered by each particle. PSO randomly initialised population of solutions, searching for the optimum solution by evolving generations. The basic steps involved in PSO operation from the initial stage to the optimum solution are depicted in Fig 2.

#### Accelerated Particle Swarm Optimisation

The APSO is a modified version of the standard PSO proposed by Yang *et al*. [41]; in APSO, convergence is accelerated by using only global best, unlike the standard PSO that uses both global best and individual best. The individual best is used for increasing diversity to obtain a quality solution, which can also be achieved using other randomness. Thus, it is not compulsory to use the individual best except in solving highly nonlinear and multimodal problems. It was found that the APSO advances the performance of the standard PSO. Compared to other variants of PSO, APSO has only two parameters: The *α* and *β* representing the learning parameters or acceleration constants (*α* ≈ *β*).

### Neural Network

**Fig 3:**
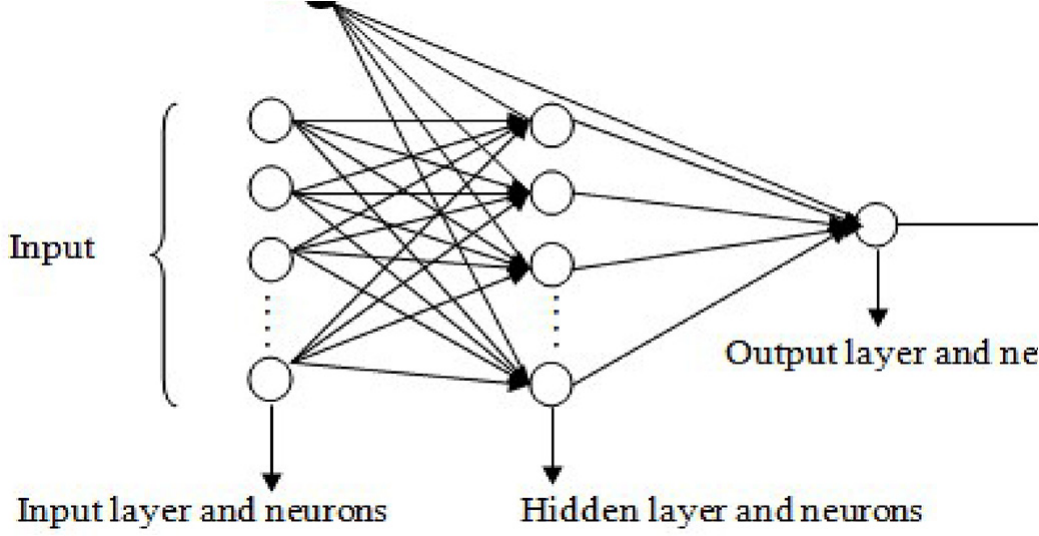
A typical structure of an ANN with input, hidden and output neurons distributed across the input, hidden and output layers respectively, where *β* is the bias.

The ANN is comprised of nodes in the input, hidden, and output layers. Nodes in the input layer feed inputs to nodes in the hidden layers, and continue in a forward direction up to the nodes in the output layer. The nodes in the input layer are configured based on the independent variables in the dataset, and the dependent variable determined the output nodes [42–43]. There can be more than one hidden layer; however, theoretical works, such as [44], argued that one hidden layer is sufficient to approximate any complex non-linear function. The number of nodes in the hidden layer is commonly realised through trial and error [45]. A typical structure of the ANN is shown in Fig 3. The ANN is an algorithm for processing information in parallel and can model complex and nonlinear associations using input–output training from datasets collected from the application domain. The intrinsic capabilities of the NN enable the algorithm to provide a nonlinear mapping of input and output vectors [43].

The NN can modify itself to perform the task if the optimal weights and bias of the NN are established [46]. There are several gradient-descent training algorithms for the optimisation of the NN weights and bias such as the Levenberg-Marquardt, backpropagation, resilient back propagation, scaled conjugate gradient, conjugate gradient with Powell-Beale restarts, Polak-Ribiere conjugate gradient, Fletcher-Reeves conjugate gradient, BFGS quasi-Newton and one-step secant algorithms. The most commonly used NN training algorithm is the backpropagation algorithm [47]. The backpropagation algorithm is a gradient method for minimising the error cost function. However, these gradient descent algorithms, are susceptible to limitations such as over-training of the NN, which could cause the training data to be overfitted and degrade the prediction accuracy. They have the possibility of being stuck in local minima, depending on the error surface shape, saturation, rate of convergence and so on [48]. Thus, the training of the NN using a HCS is ideal because the limitations of the gradient descent algorithms can be eliminated.

## The Organization of the Petroleum Exporting Countries’ CO_2_ Emissions Dataset

The dataset for the OPEC CO_2_ emissions from the consumption of petroleum in million metric tons (mmt) from 1980 to 2011 was collected from [49], a credible source of energy data [50]. The data are collected yearly, in view of the fact that the data are available on a yearly basis. Data availability determined the collection period and frequency [51]. The data is comprised of the 12 OPEC countries’ CO_2_ emissions and the total OPEC CO_2_ emissions. The columns and rows of the dataset are 13 and 32 respectively. The basic statistics of the dataset are presented in Table 1 showing the maximum, minimum, mean and standard deviation (SD) for each OPEC country CO_2_ emissions dataset including the OPEC for the data collection period.

**Table 1:** Basic descriptive statistics of the OPEC countries CO_2_ emissions dataset.

Country	No. Years	Minimum	Maximum	Mean	SD
OPEC	32	16.63	43.71	26.2064	6.74531
Algeria	32	2.75	12.80	5.5004	2.84066
Angola	32	11.57	30.78	17.8465	5.30320
Ecuador	32	82.41	284.57	168.7033	50.64198
Iran	32	29.07	120.63	62.0111	22.28333
Iraq	32	12.23	61.98	31.8385	15.86746
Kuwait	32	14.14	42.43	28.0091	8.55353
Libya	32	23.85	45.39	36.5936	5.39421
Nigeria	32	1.48	18.39	6.3378	4.75290
Qatar	32	88.52	323.88	179.2568	65.35749
Saudi Arabia	32	10.61	95.67	49.6525	20.89947
United Arab Emirates	32	53.19	104.07	66.6684	15.26779
Venezuela	32	356.04	1159.76	678.6245	214.79416

**Fig 4:**
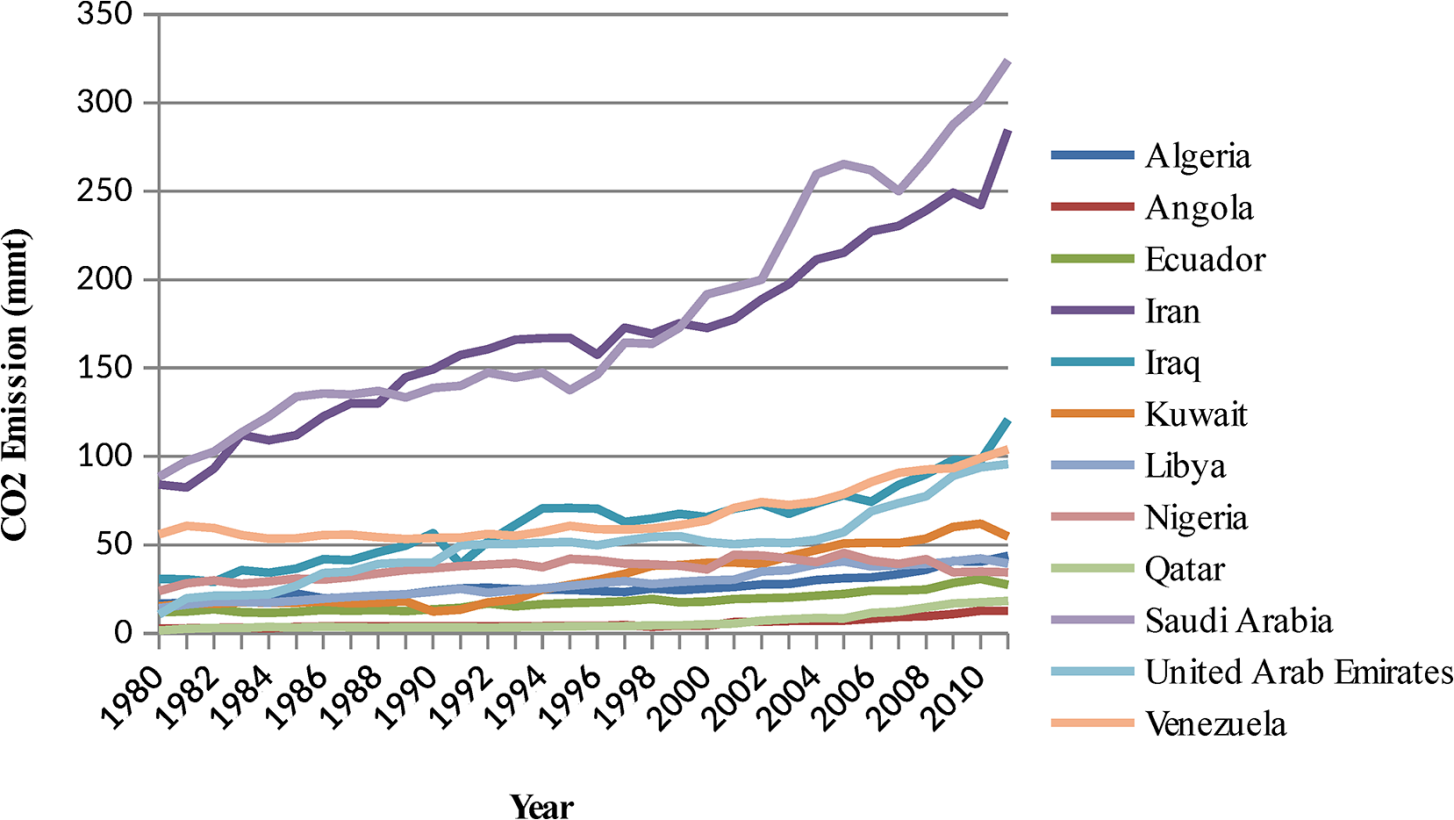
The pattern of CO_2_ emissions from petroleum consumption in OPEC countries (1980–2011).

The OPEC CO_2_ emission is the dependent variable, whereas the CO_2_ emissions from the 12 member countries of the OPEC, as shown in Fig 4, are the independent variables representing the inputs. Therefore, the CO_2_ emissions of the 12 OPEC countries are used as the inputs to predict the OPEC CO_2_ emissions from petroleum consumption.

The dataset was normalised to a range of [–1,1] using Eq (7) to improve prediction accuracy and convergence speed [52].

$$n_{o}=\frac{k_{i}-x_{\text{min}}}{p_{\text{max}}-x_{\text{min}}}$$ (7)

Where *n*_*o*_ = normalise dataset, *k*_*i*_ = raw dataset, *x*_min_ = minimum value of the dataset *and p*_max_ = maximum value of the dataset. The OPEC CO_2_ emissions dataset was analysed using correlation to investigate the relationship between dependent variables and between dependent and independent variables. Successful prediction requires that the variables involved in the task be positively related [53]. Table 2 is the correlation matrix of the variables involved in the prediction. It was found that the relationships among the variables are positively related. This makes the variables suitable for the prediction.

**Table 2:** An inter correlation matrix showing relationships among the 12 member countries CO_2_ emissions from petroleum consumption as well as the relationship between OPEC CO_2_ emissions and each member country

	Algeria	Angola	Ecuador	Iran	Iraq	Kuwait	Libya	Nigeria	Qatar	Saudi Arabia	United Arab Emirates	Venezuela
Algeria												
Angola	0.961**											
Ecuador	0.950**	0.944**										
Iran	0.953**	0.907**	0.939**									
Iraq	0.925**	0.883**	0.923**	0.958**								
Kuwait	0.875**	0.890**	0.957**	0.900**	0.885**							
Libya	0.899**	0.880**	0.943**	0.958**	0.908**	0.946**						
Nigeria	0.439^*^	0.341	0.499**	0.625**	0.582**	0.513**	0.681**					
Qatar	0.960**	0.985**	0.933**	0.888**	0.861**	0.885**	0.854**	0.273				
Saudi Arabia	0.950**	0.952**	0.949**	0.955**	0.901**	0.944**	0.956**	0.496**	0.940**			
United Arab Emirates	0.951**	0.897**	0.938**	0.963**	0.945**	0.855**	0.898**	0.548**	0.885**	0.898**		
Venezuela	0.924**	0.974**	0.937**	0.872**	0.853**	0.912**	0.868**	0.322	0.975**	0.936**	0.844**	
OPEC	0.968**	0.950**	0.972**	0.987**	0.955**	0.946**	0.969**	0.562**	0.935**	0.984**	0.953**	0.929**

**Correlation is significant at the 0.01 level (2-tailed).

## The Design of the Proposed Hybrid Cuckoo Search Neural Network (HCSNN)

**Fig 5:**
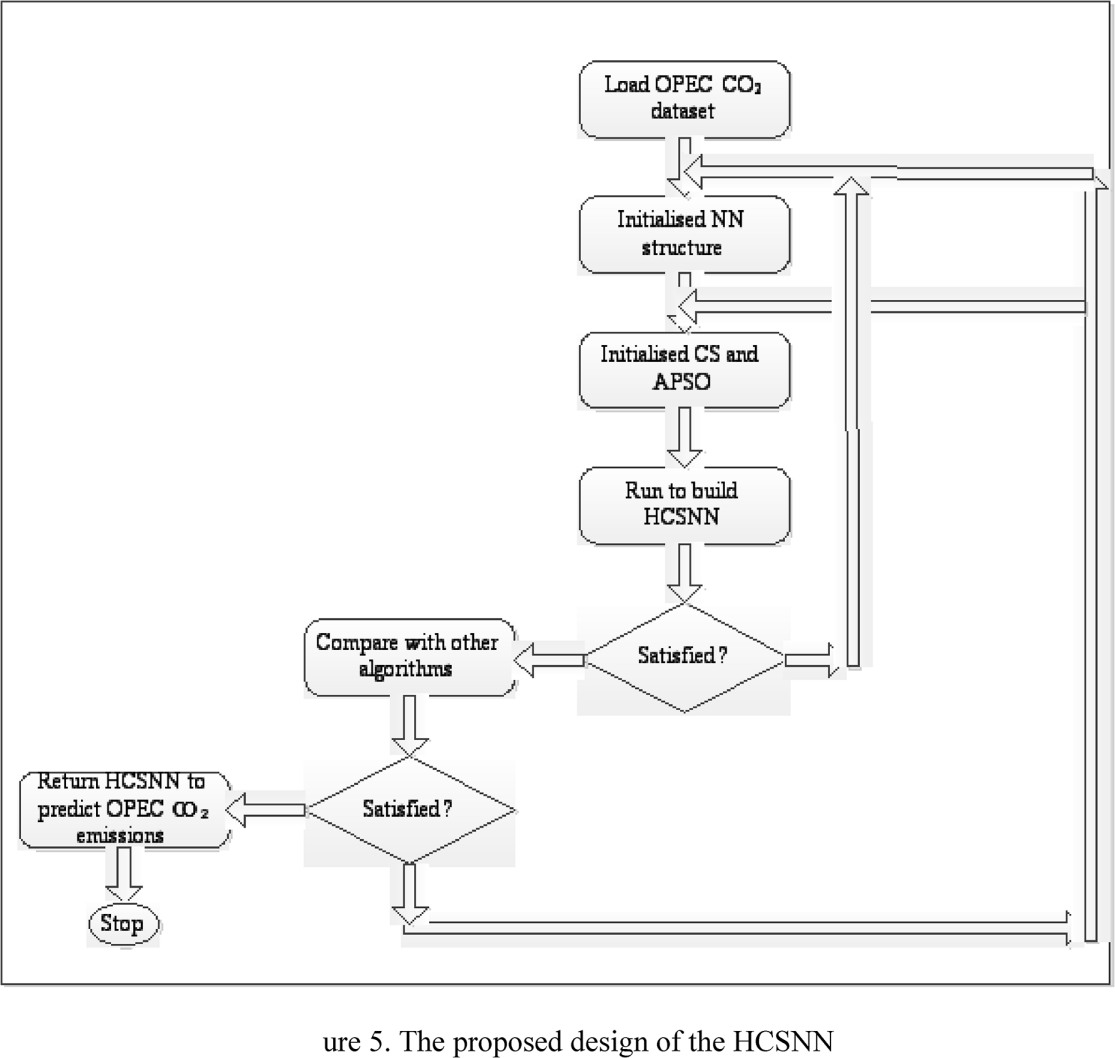
The proposed design of the HCSNN.

The major components of the proposed method are presented in a flowchart in Fig 5. The major stages comprised of the dataset, modeling, and evaluation. In the proposed approach, CS is hybridised with APSO to build the HCS. In the proposed HCS, communication capability of the cuckoo births have been improved by introducing APSO to search for a better location in which the optimal nest can share information with the cuckoo. Thus, the HCS chooses the optimal nest among all the nests via lévy flight, unlike in the standard CS (refer to section 2.1). The HCS performs the search using Eq (8) [40–41]. The standard Equation of the CS is given in Eq (6). Eq (9) is the proposed equation in which the velocity vector *v*_*i*_^*t*+1^ is taken from Eq (9) which is the standard Eq of the APSO [41]. The proposed Eq (9) is derived from Eq (6) and Eq (8).

$$v_{i}{}^{t+1}=v_{i}{}^{t}+\alpha \varepsilon_{n}+\beta \left(g^{*}-x_{i}{}^{t}\right)$$ (8)

$$x_{i}{}^{t+1}=x_{i}{}^{t}+\alpha ⊕levy\left(\lambda \right)+v_{i}{}^{t+1}$$ (9)

Where *v*_*i*_^*t*+1^ is the velocity vector, *v*_*i*_^*t*^ and *x*_*i*_^*t*^ are positions vector for the particle, and *ε*_*n*_ represents the random vector typically drawn from [0,1]. The current global best is represented by *g* ^*^. The mean square error (MSE) is chosen as the objective function because the HCSNN performance is to be compared with other meta-heuristic algorithms for evaluation purposes. The MSE is better than other performance indicators such as normalised mean square error, sum of square error, etc. in comparing performance of different algorithms on the same dataset [43].

To really assess the performance of the proposed method, the HCSNN was experimented across several training and test datasets with varying data partition ratios (training–testing); given that training data has an effect on the performance of the prediction [54], a similar practice was used in [55]. Therefore, five different data partition ratios were used in this study and each was run 10 times because meta-heuristic algorithms are required to be run more than once to compute the mean, best and the worst results as meta-heuristic algorithms are not deterministic. The best solution is typically realised from multiple execution of the algorithm [56]. The input neurons of the ANN are set to 12 in view of the fact that the independent variables in the dataset are 12, and the output neuron is set to 1 because only one dependent variable is used (refer to section 3). The hidden neurons were fixed to 5 as suggested by experimental trials. There are many activation functions but *tanh* is preferred in the hidden layer of ANN for solving prediction problems [57], and *linear* in the output layer as recommended by Beale *et al*. [58]. The objective of the HCS is to train the ANN to optimise its weights and bias.

**Fig 6:**
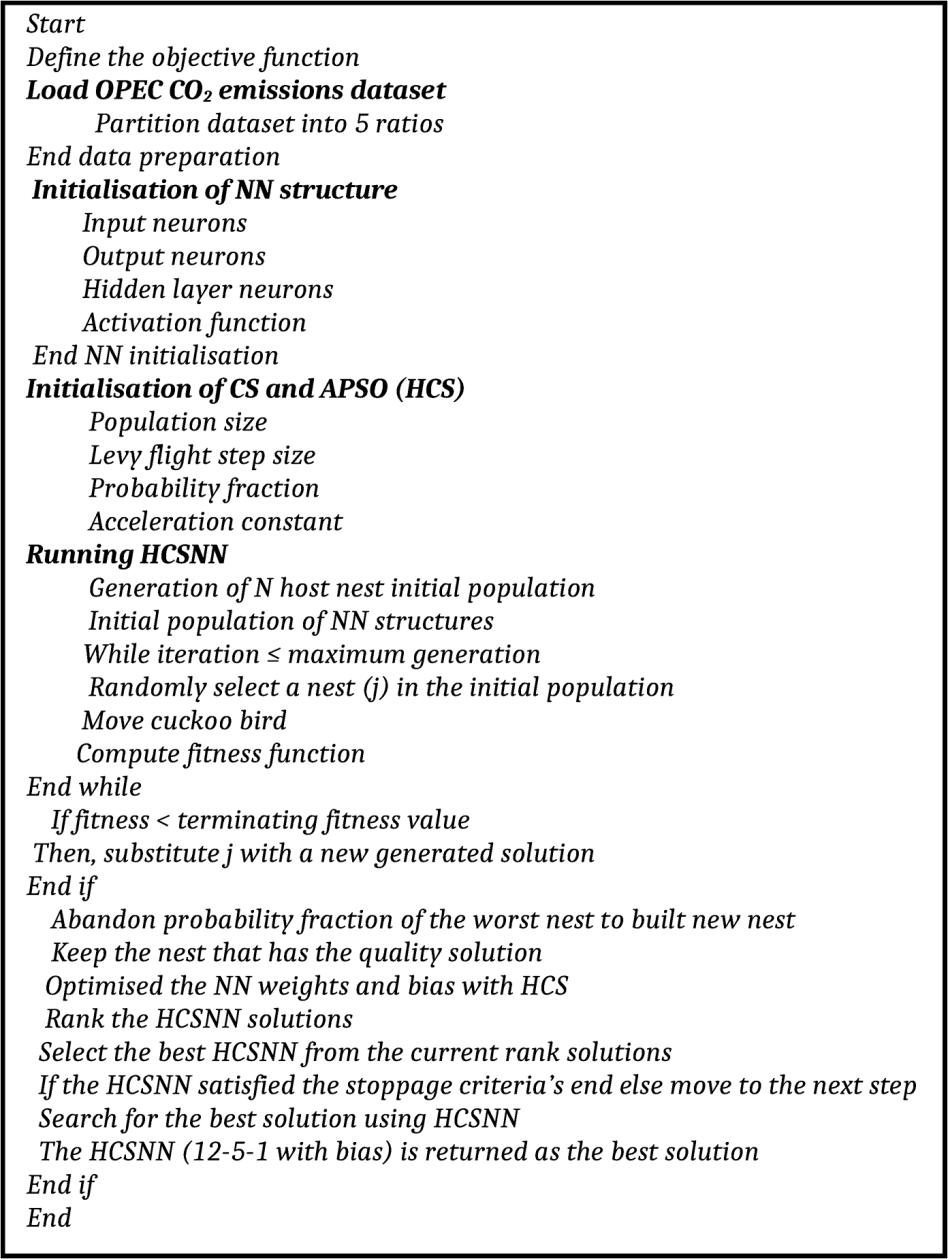
Pseudo-code of the proposed HCSNN.

Running HCS requires initialisation to start running. The HCS, like other meter-heuristic algorithms, requires the setting of parameter values. There is no systematic, universally agreed method of getting the best settings of meta-heuristic algorithms [28]. In this study, we adopted the parameters *Pa* = 0.25, α_1_ = 1, *n* = 25 [36], *α* = 0.7 [41]. The proposed HCSNN was run for a maximum of 1000 generations to build a HCSNN-with-bias for the prediction of OPEC CO_2_ emissions. The pseudo-code of the HCSNN proposed in the research is presented in Fig 6. For the purpose of evaluating the effectiveness of our method, we used standard CS, GA, PSO, ABC to optimise the weights and bias of the ANN to build CSNN, GANN, PSONN, ABCNN for the prediction of OPEC CO_2_ emissions. The results of the proposed and comparative methods are compared.

## Results and Discussion

The numerical results of the experiments conducted using the OPEC CO_2_ emissions datasets are shown and discussed in this section. Experimental simulation analysis shows that it is possible to predict OPEC CO_2_ emissions in 3, 6, 9, 12, and 16 years using the proposed HCSNN.

### Sensitivity of the ANN and CS configuration parameters

The results of the experimental trials to investigate several configurations of the ANN with regard to the variety of CS parameter settings are presented in Table 3. The experiments are repeated for different number of hidden layer neurons starting from 2 with an increment of 1 up to 7. The experiment was stopped at 7 hidden layer neurons in all the trials because it was observed that the MSE started reducing from 6 hidden neurons. A similar phenomenon was observed by Uzer *et al*. [59] in their experiments. All the experiment trials conducted has proven that 5 hidden neurons were the best for the ANN. The sensitivity of the CS parameters as shown in Table 3 has influence on the ANN performance. This is not surprising because meta-heuristic algorithms are sensitive to parameter settings, though CS require the setting of only *P*_*a*_ and α parameters [60]. Among the CS parameters used in the experiment trials, the values suggested by Yang and Deb [36] were found to be the best settings for the CS. Many literature [61–63] adopted the CS settings proposed by Yang and Deb [36] for the execution of CS because of their performance.

**Table 3:** Experiments with several ANN configurations and CS parameters.

CS parameters	*Pa* = 0.25, α_1_ = 0.15	*Pa* = 0.5, α_1_ = 1.3	*Pa* = 0.11, α_1_ = 0.8	*Pa* = 0.25, α_1_ = 1
Hidden neurons	MSE	MSE	MSE	MSE
2	0.007318	0.087127	0.0025371	0.00067113
3	0.0045251	0.006345	0.0014357	0.00027865
4	0.0032145	0.001734	0.0001924	0.00009251
5	0.0005110	0.000277	0.0001136	0.00000345
6	0.0029239	0.076812	0.0005643	0.00007452
7	0.0067871	0.047116	0.0007241	0.00008741

### Comparing HCSNN with the Standard CSNN and APSONN

The experiments were implemented using MATLAB R2012b on a machine (Intel Core 2 Quad CPU 2.33GHz, RAM 2GB, 32-bit operating system). The source code can directly be requested from abdullahdirvi@gmail.com. The comparison between the proposed HCSNN and the basic CSNN and APSONN were first performed. Subsequently, the proposed HCSNN is compared with other established algorithms (GANN and ABCNN). In Chiroma *et al*. [64], their proposed meta-heuristic algorithm method of modelling oil consumption was compared with other meta-heuristic algorithms. Tables 4–7 summarised the simulation results; the first column is the data partition ratio, whereas the second, third, and fourth columns are the mean, best, and worst results, respectively. The results were obtained for each of the algorithms after the experiments on both training and test OPEC CO_2_ emissions datasets.

**Table 4:** Comparing HCSNN, CSNN, and APSONN training time (seconds) on the OPEC CO_2_ emissions training dataset.

		CSNN			HCSNN			APSONN	
Data partition	Mean	Best	Worst	Mean	Best	Worst	Mean	Best	Worst
90–10	134.1577	133.15	134.99	8.3048	**7.30**	9.31	100.6197	100.17	101.07
80–20	42.2520	40.84	43.55	15.0016	**13.81**	16.05	103.3667	102.90	103.83
70–30	33.2189	32.04	34.39	3.4986	**1.97**	5.18	102.9460	102.48	103.41
60–40	13.5848	12.45	14.59	0.3799	**0.37**	0.39	102.8002	102.26	103.30
50–50	2.8691	2.16	3.04	0.3794	**0.36**	0.39	103.1377	102.64	103.63

**Table 5:** Comparing HCSNN, CSNN, and APSONN accuracy (MSE) on the OPEC CO_2_ emissions training dataset.

		CSNN			HCSNN			APSONN	
Data partition	Mean	Best	Worst	Mean	Best	Worst	Mean	Best	Worst
90–10	0.000012130	0.0000088	0.0000125	0.000014080	**0.0000119**	0.0000219	0.000916878	0.0009169	0.0009169
80–20	0.000010456	0.0000096	0.0000106	0.000012367	**0.0000094**	0.0000127	0.000576254	0.0005763	0.0005763
70–30	0.000010010	0.0000069	0.0000108	0.000020821	**0.0000028**	0.0000323	0.000880278	0.0008803	0.0008803
60–40	0.000011747	0.0000096	0.0000123	0.000000132	**2.79E-9**	0.0000006	0.000054600	0.0000546	0.0000546
50–50	0.000107377	0.0000054	0.0002189	0.000000002	**2.15E-10**	0.0000015	0.000513349	0.0005133	0.0005133

**Table 6:** Comparing HCSNN, CSNN, and APSONN test time (seconds) on OPEC CO_2_ emissions test datset.

		CSNN			HCSNN			APSONN	
Data partition	Mean	Best	Worst	Mean	Best	Worst	Mean	Best	Worst
90–10	0.8130	0.65	0.98	0.4072	**0.25**	0.51	102.8781	102.38	103.34
80–20	3.0912	2.07	3.77	1.4034	**0.74**	2.19	102.1031	101.65	102.56
70–30	2.1920	1.89	2.48	1.7691	**1.27**	2.52	102.9715	102.52	103.43
60–40	100.9103	99.80	102.02	0.3709	**0.37**	0.38	102.8002	102.26	103.30
50–50	28.3647	27.23	29.50	0.3845	**0.35**	0.46	101.1676	100.69	101.63

**Table 7:** Comparing HCSNN, CSNN, and APSONN accuracy (MSE) on OPEC CO_2_ emissions test dataset.

		CSNN			HCSNN			APSONN	
Data partition	Mean	Best	Worst	Mean	Best	Worst	Mean	Best	Worst
90–10	0.000007990	0.0000014	0.0000146	0.000010800	**0.00000108**	0.0000108	0.000039400	0.0000394	0.0000394
80–20	0.000015698	0.0000100	0.0000280	0.000038002	**0.0000075**	0.0000955	0.000347767	0.0003478	0.0003478
70–30	0.000018227	0.0000091	0.0000370	0.000014176	**0.0000084**	0.0000251	0.001125384	0.0011254	0.0011254
60–40	0.000010518	0.0000098	0.0000106	0.000000318	**2.79E-9**	0.0000006	0.000054600	0.0000546	0.0000546
50–50	0.000011409	0.0000097	0.0000116	0.000000380	**2.15E-10**	0.0000015	0.001395282	0.0013953	0.0013953

Tables 4–7 reported the performance of the proposed HCSNN, CSNN, and APSONN on the training and test OPEC CO_2_ emissions datasets. The HCSNN was found to converge to the optimal solution faster than the CSNN, and APSONN on both training and test datasets. Therefore, the proposed HCSNN can be considered as the best algorithm because the best algorithm converges to the best solution within a short period of time [28, 65]. The proposed HCSNN has improved the performance of the CSNN and APSONN prediction methods. This signifies that the proposed HCSNN has the capability of providing a better solution in a short period of time. The performance advances made by the proposed HCSNN over the standard CSNN and APSONN could probably be achieved because of the hybridisation of the standard CS and APSO, which improves the communication capability of the cuckoos to search for a better location where the optimal nest can share information with the cuckoo; hence, it improves the performance of the CS and the APSO to converge to the optimal solution very fast.

### Comparing Performance of HCSNN, GANN, and ABCNN

Comparing a proposed method based on meta-heuristic algorithm to other meta-heuristic algorithms [66] is required. The proposed HCSNN performance was compared with established meta-heuristic algorithms, that is, GA, and ABC [29]. Thus, GANN and ABCNN were applied for the prediction of OPEC CO_2_ emissions from petroleum consumption. The experimental results of the study are summarised in Tables 8–11.

**Table 8:** GANN and ABCNN training time (seconds) on OPEC CO_2_ emissions training dataset.

		GANN			ABCNN	
Data partition	Mean	Best	Worst	Mean	Best	Worst
90–10	5.6703	5.65	5.70	246.3318	245.26	247.41
80–20	5.5824	5.56	5.61	241.2392	240.16	242.33
70–30	5.5341	5.51	5.56	241.9189	240.85	242.99
60–40	5.6439	5.62	5.67	243.1096	242.03	244.18
50–50	5.5498	5.53	5.57	241.0873	240.01	242.18

**Table 9:** GANN and ABCNN accuracy (MSE) on OPEC CO_2_ emmissions training dataset.

		GANN			ABCNN	
Data partition	Mean	Best	Worst	Mean	Best	Worst
90–10	0.013437393	0.0130828	0.0141054	0.001295257	0.0012946	0.0012953
80–20	0.005834083	0.0032442	0.0099324	0.001671239	0.0016712	0.0016712
70–30	0.006426911	0.0064269	0.0064269	0.000631877	0.0006319	0.0006319
60–40	0.004693395	0.0046934	0.0046934	0.000684235	0.0006641	0.0006893
50–50	0.003083721	0.0030837	0.0030837	0.000286135	0.0002860	0.0002863

**Table 10:** GANN and ABCNN test time (seconds) on OPEC CO_2_ emmissions test dataset.

		GANN			ABCNN	
Data partition	Mean	Best	Worst	Mean	Best	Worst
90–10	5.5245	5.50	5.55	245.7797	244.70	246.84
80–20	5.5057	5.48	5.53	240.1158	239.05	241.18
70–30	5.4879	5.46	5.51	244.5283	243.47	245.59
60–40	5.6292	5.61	5.65	248.4572	247.38	249.53
50–50	5.5806	5.56	5.60	244.2724	243.18	245.36

**Table 11:** GANN and ABCNN accuracy (MSE) on OPEC CO_2_ emmsisions test dataset.

		GANN			ABCNN	
Data partition	Mean	Best	Worst	Mean	Best	Worst
90–10	0.076082131	0.0751107	0.0771107	0.002150212	0.0021265	0.0021857
80–20	0.061608778	0.0616088	0.0616088	0.000978319	0.0009783	0.0009783
70–30	0.052073117	0.0520731	0.0520731	0.000734849	0.0007348	0.0007348
60–40	0.043550760	0.0435508	0.0435508	0.000838752	0.0008388	0.0008388
50–50	0.035446093	0.0354461	0.0354461	0.002150212	0.0021265	0.0021857

The performance of the GANN and ABCNN on training and testing OPEC CO_2_ emissions datasets are reported in Tables 8–11. A comparison of the performance of the proposed HCSNN (see Tables 4–7, bold) with that of GANN and ABCNN (Tables 8–11) on training and test dataset showed that the proposed HCSNN can provide better accuracy and convergence speed than the GANN and ABCNN on both training and test dataset. Those results have further validated the effectiveness and robustness of the HCSNN in the prediction of OPEC CO_2_ emissions. The performance of the HCSNN can probably be attributed to the CS in twofold: (1) The CS striking balance between local and global search; (2) the CS requires few parameters to run successfully unlike GA and ABC that require more parameters settings than the CS.

### Predicted vs. Actual OPEC CO_2_ Emissions from Petroleum Consumption

**Fig 7:**
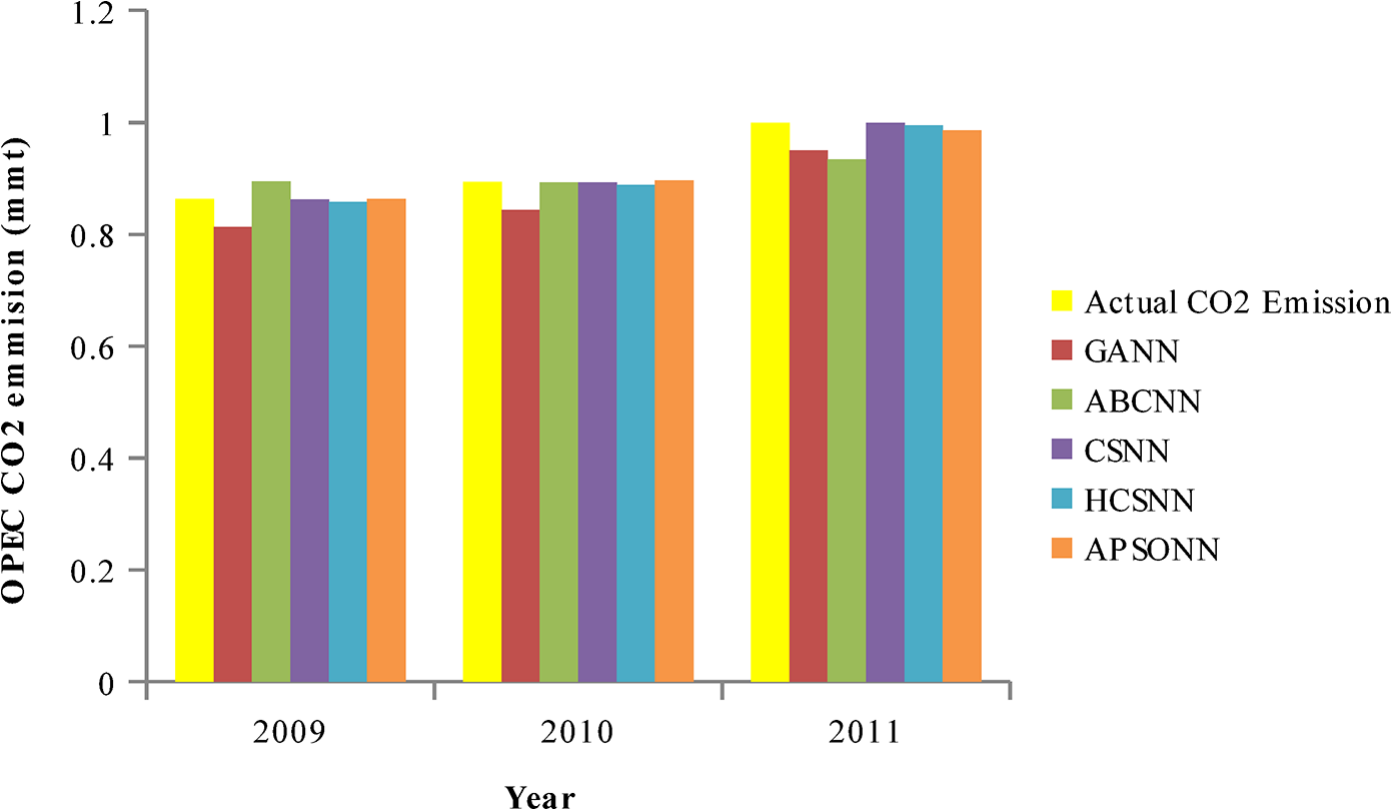
Predicted vs. actual OPEC CO_2_ emissions (90–10).

**Fig 8:**
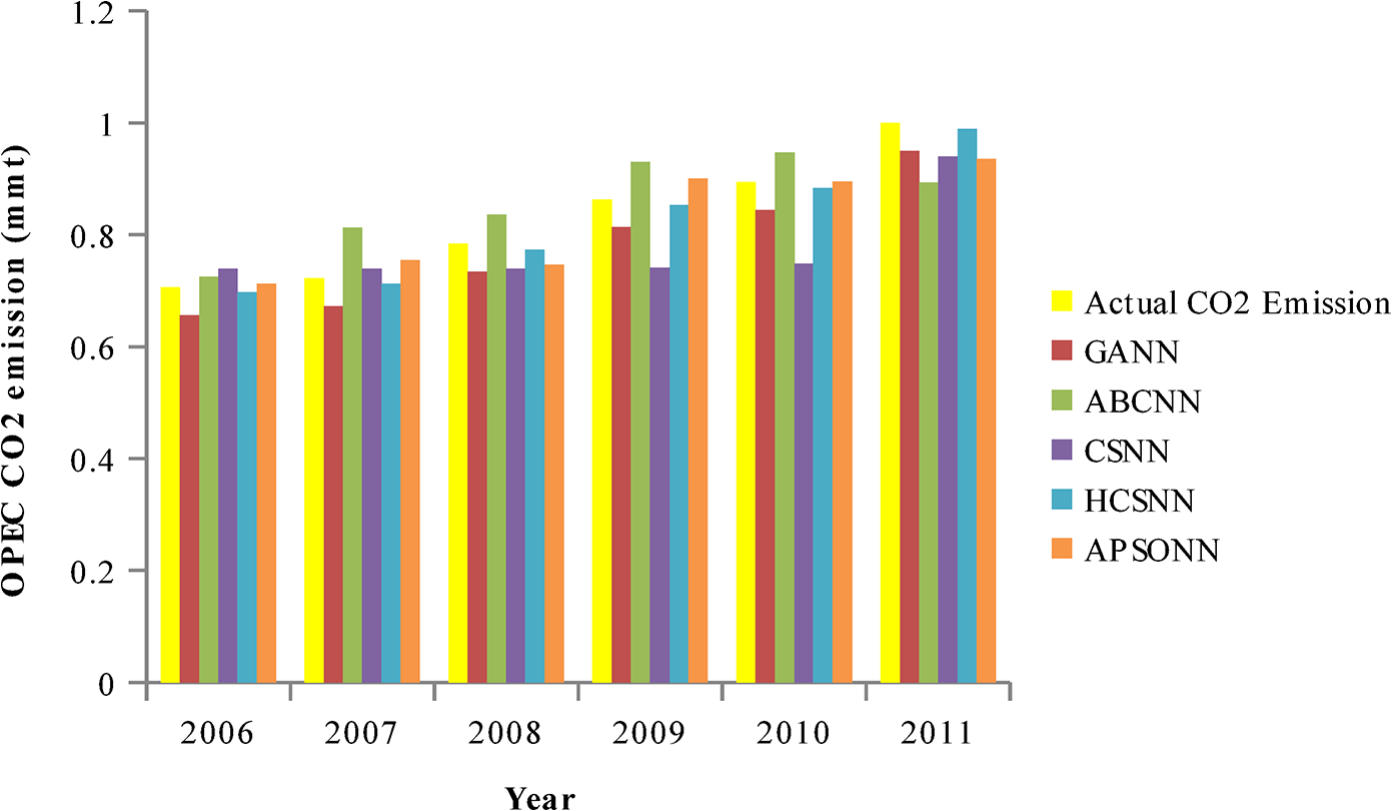
Predicted vs. actual OPEC CO_2_ emissions (80–20).

**Fig 9:**
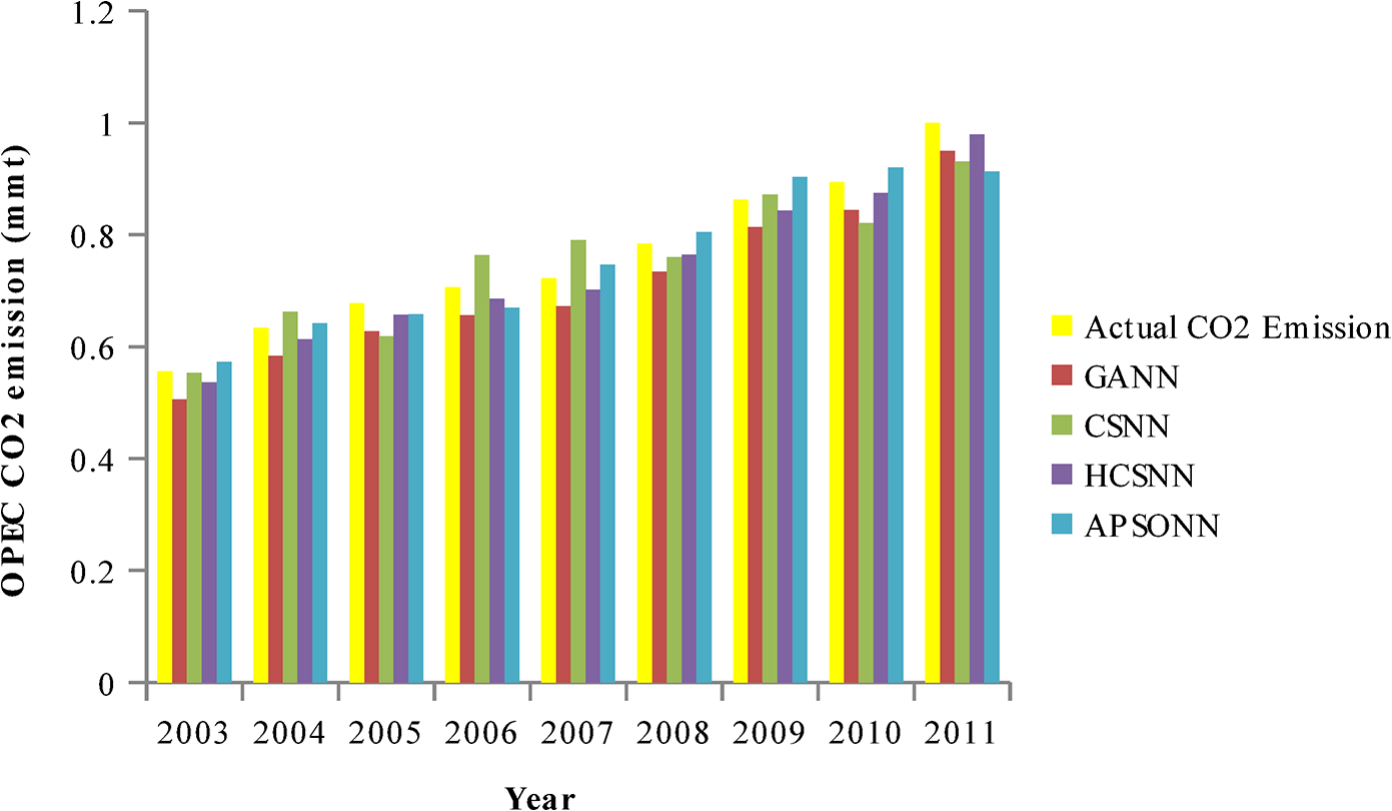
Predicted vs. actual OPEC CO_2_ emissions (70–30).

**Fig 10:**
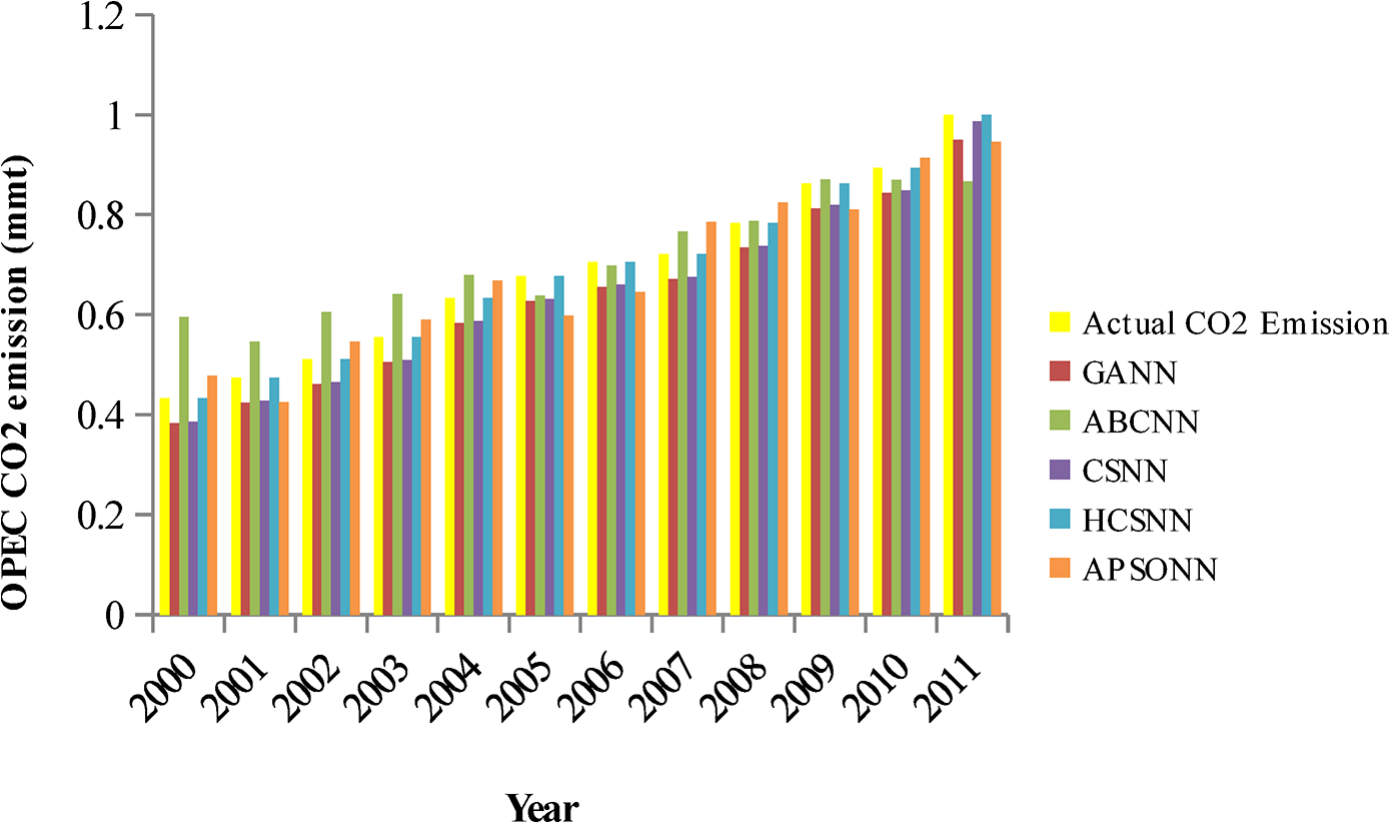
Predicted vs. actual OPEC CO_2_ emissions (60–40).

**Fig 11:**
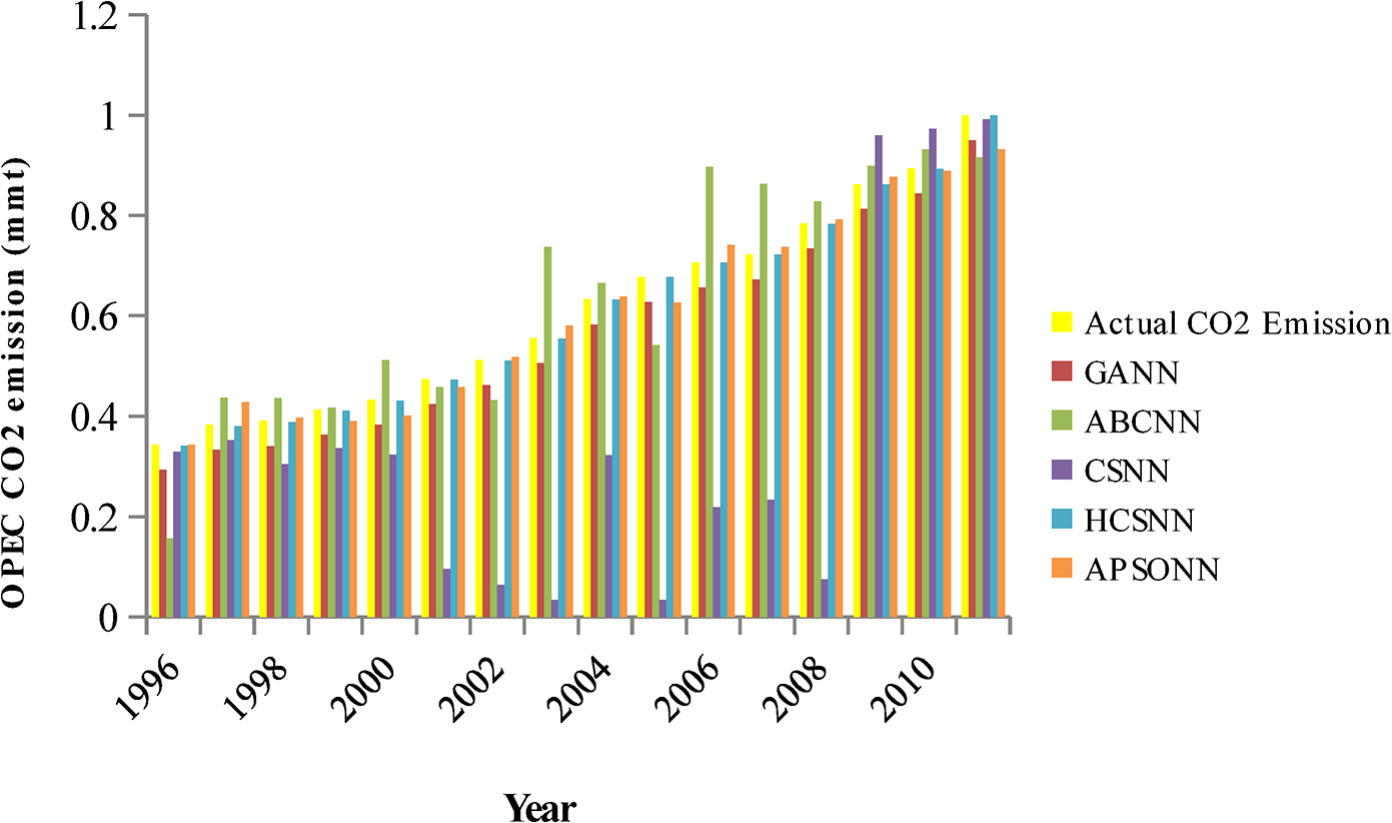
Predicted vs. actual OPEC CO_2_ emissions (50–50).

The pattern of the actual OPEC CO_2_ emissions and the predicted ones by the algorithms (HCSNN, GANN, ABCNN, and APSONN) are depicted in Figs 7–11. The prediction is based on the OPEC CO_2_ emission test dataset reserved for evaluation purpose. The prediction is for 3, 6, 9, 12, and 16 years, respectively. The performance indicators are in Tables 4–7 and Tables 8–11.

It can be observed in Figs 7–11, that the OPEC CO_2_ emissions predicted by the proposed HCSNN is more fit to the actual OPEC CO_2_ emissions than the other comparison algorithms. In 3, 6, 9, and 12 year predictions (Figs 7–10) the other compared algorithms also fitted close to the actual OPEC CO_2_ emissions except for GANN. The abolition of previous knowledge by GA could possibly be responsible for the low performance of the GANN. In 16 year predictions, ABCNN and CSNN move further away from the actual OPEC CO_2_ emissions. This has clearly shown that the algorithms are not robust as the number of the predicted years increases. Thus, ABCNN, GANN, and CSNN are not consistent in their performance. The APSONN performance is consistent. However, the proposed HCSNN is consistent and more accurate than the APSONN in the prediction of OPEC CO_2_ emissions, as the HCSNN has maintained similar performance throughout the prediction periods. Therefore, the HCSNN is robust, accurate and fast in the prediction of OPEC CO_2_ emissions. The optimum solution for application in solving real world problem is required to be robust in addition to accuracy and convergence speed as argued by Yang and Deb [28].

## Policy Implications

In view of the fact that protection against global warming requires a holistic approach, accurate prediction of OPEC CO_2_ emissions from petroleum consumption can give member countries a better estimate of CO_2_ emissions expected in the future, thus allowing OPEC to create a robust CO_2_ emissions policy involving the 12 member countries. OPEC members are skeptical, however, about the reduction of CO_2_. This is because it may increase the price of oil to consumers, thus decreasing demand from developed countries, which accounts for 60% of total oil consumption in the world [67]. This can obstruct development and decline in revenue generation in OPEC countries, given that the main source of government revenue in OPEC countries is the sale of petroleum.

Reducing oil consumption means reducing CO_2_ emissions. If some OPEC countries are reducing CO_2_ emissions while others are not, surely it can affect other members’ CO_2_ emissions (see Table 2). Thus, a holistic approach is required by all the member countries to put measures in place that will drastically reduce CO_2_ emissions in all the countries if meaningful results are to be achieved. However, reducing oil consumption must be done with a caution given that oil consumption is significantly positively related to economic development as described in [3].

Since OPEC members are developing countries, the reduction of CO_2_ must be done with precautions in order not to slow down economic development and generation of revenue. An accurate prediction of OPEC CO_2_ emissions can serve as a reference point for an OPEC secretariat to propagate the reorganisation of economic development in member countries with the view of managing CO_2_ emissions. Evidence of CO_2_ emission dangers can easily be used to convince member countries to embark on economic development that can result to minimal petroleum consumption and reduced CO_2_ emissions. In view of the economic implications of reducing CO_2_ emissions, reduction of the CO_2_ emissions in OPEC countries must be enforced with caution. Considering the contributions of OPEC countries to global warming, it is significant for OPEC to adapt its policies on climate change that can enforce stringent measures for the members to adopt an energy-efficient economy.

Meng *et al*. [18] argued that the CO_2_ emissions emanating from countries that are developing has attracted unprecedented attention to economic development and the increasing consumption of fossil energy consumption. The Efforts been taken by the developing countries in monitoring and controlling the emissions of CO_2_ have become a premise to further maintain the Kyoto benchmark on climate change alleviation.

The future prediction of CO_2_ emission is one of the major factors for the management, control and modification of a state of the art policies related to CO_2_ emissions [18, 21]. The management and control of the emissions of CO_2_ drastically reduce the negative effects of global warming [10–11].

The limitations of our study: The prediction was performed based on historical data. As such, future predicted CO_2_ emissions can be affected by a prolonged wars or famines that can bring down the economic growth. As a result, the emission of CO_2_ emissions can be decreased in the future. Also, the data were collected on yearly frequency. Therefore, the prediction horizon is limited to yearly basis.

## Conclusions

This paper proposed a method for the prediction of OPEC CO_2_ emissions based on CS, ANN, and APSO to improve accuracy and convergence speed. The dataset required for the modelling was collected from the Energy Information Administration. We built a HCSNN model to predict OPEC CO_2_ emissions. Intensive experiments were conducted with HCSNN and other meta-heuristic algorithms such as CSNN, PSONN, GANN, and ABCNN to predict OPEC CO_2_ emissions in 3, 6, 9, 12, and 16 years. Comparative results indicated that the proposed HCSNN advanced the prediction accuracy and convergence speed of the comparison meta-heuristic algorithms in all the years. Accurate and timely prediction of OPEC CO_2_ emissions can allow OPEC member countries to accurately adapt OPEC policies related to climate change. This is because the more the prediction accuracy of CO_2_ emissions, the more the accuracy of the decision to be taken on climate change policies, hence, reducing the contributions of the OPEC countries to global warming. In the future, the method presented in this study will be modified to investigate the effectiveness of the method in the estimate of CO_2_ loss from the streams [68]. The method presented in this research could easily be implemented into software to develop a decision system capable of advising OPEC policy makers with predicted values of CO_2_ emissions.

## Supporting Information

S1 Supporting Information OPEC CO2 emssions Dataset. (DOCX)

S2 Supporting Information source code.zip. (ZIP)
