# Supplementary material for: Global Warming: Predicting OPEC Carbon Dioxide Emissions from Petroleum Consumption Using Neural Network and Hybrid Cuckoo Search Algorithm
Source: PLoS One. 2015 Aug 25;10(8):e0136140. doi: 10.1371/journal.pone.0136140 (PMC4549267; doi:10.1371/journal.pone.0136140)
Supplement: S1 Supporting Information — (DOCX) [file pone.0136140.s001.docx]

Dataset

|  | Algeria | Angola | Ecuador | Iran | Iraq | Kuwait | Libya | Nigeria | Qatar | Saudi Arabia | United Arab Emirates | Venezuela | OPEC |
| --- | --- | --- | --- | --- | --- | --- | --- | --- | --- | --- | --- | --- | --- |
| 1980 | 16.62676 | 2.87925 | 11.80931 | 84.01607 | 30.74789 | 15.27971 | 14.1419 | 23.85056 | 1.4769 | 88.51775 | 10.61193 | 56.08262 | 356.0407 |
| 1981 | 17.30865 | 2.88961 | 12.40532 | 82.41158 | 30.45269 | 16.6508 | 16.32889 | 28.1587 | 2.5645 | 97.33777 | 19.82755 | 60.6563 | 386.9924 |
| 1982 | 19.54381 | 3.03153 | 13.54402 | 93.26594 | 29.06612 | 16.67801 | 17.64394 | 29.90337 | 2.93853 | 102.7613 | 21.33076 | 59.55281 | 409.2601 |
| 1983 | 18.518 | 3.16293 | 11.93822 | 112.2251 | 35.60478 | 17.52719 | 17.72622 | 28.12166 | 3.00403 | 113.4058 | 21.45295 | 55.51833 | 438.2052 |
| 1984 | 20.97897 | 2.7468 | 11.57018 | 109.0549 | 34.31829 | 17.30761 | 17.19563 | 29.20534 | 3.72153 | 122.5082 | 22.00752 | 53.46551 | 444.0804 |
| 1985 | 22.48641 | 3.56522 | 12.10068 | 112.012 | 36.46165 | 17.38059 | 18.28998 | 30.93902 | 3.21663 | 133.7724 | 26.81797 | 53.62322 | 470.6657 |
| 1986 | 19.85978 | 3.81689 | 13.16425 | 122.6428 | 41.9345 | 17.82297 | 19.48635 | 30.23099 | 3.55968 | 135.594 | 34.12145 | 55.55683 | 497.7906 |
| 1987 | 20.36317 | 3.76925 | 12.75827 | 130.0307 | 41.22126 | 16.79285 | 20.54429 | 31.65005 | 3.51346 | 134.9258 | 34.81956 | 55.79833 | 506.187 |
| 1988 | 20.80265 | 3.85724 | 13.04313 | 129.9882 | 45.85545 | 17.27896 | 21.42752 | 33.89413 | 3.30935 | 137.1335 | 39.12168 | 54.24588 | 519.9577 |
| 1989 | 22.06786 | 3.89101 | 12.45369 | 144.8085 | 49.54608 | 17.99741 | 22.13603 | 35.63467 | 3.40992 | 133.304 | 40.0194 | 53.19119 | 538.4597 |
| 1990 | 23.9442 | 3.79343 | 13.53151 | 149.2936 | 56.65809 | 12.2302 | 23.55016 | 36.68968 | 3.44518 | 138.7112 | 40.08528 | 53.95076 | 555.8833 |
| 1991 | 25.26898 | 3.94864 | 14.43776 | 157.4231 | 39.00059 | 13.28322 | 25.17348 | 37.88292 | 3.41474 | 139.8497 | 49.78306 | 54.19503 | 563.6612 |
| 1992 | 25.68284 | 4.05688 | 16.79832 | 160.6775 | 51.89294 | 17.45847 | 22.94517 | 38.90683 | 3.32033 | 147.4648 | 50.41358 | 56.23294 | 595.8506 |
| 1993 | 24.88502 | 3.99579 | 15.19884 | 165.9298 | 61.34915 | 19.16674 | 24.37682 | 39.73012 | 3.43883 | 144.4776 | 50.56216 | 55.08548 | 608.1963 |
| 1994 | 24.63918 | 3.94821 | 16.58074 | 166.8822 | 70.6148 | 24.83035 | 25.27225 | 37.34792 | 3.527 | 147.4056 | 51.18811 | 57.4364 | 629.6728 |
| 1995 | 24.53574 | 4.24089 | 17.09633 | 167.143 | 70.77995 | 27.36694 | 26.81146 | 42.18761 | 3.82808 | 137.5434 | 51.64783 | 60.74364 | 633.9249 |
| 1996 | 23.99515 | 3.95317 | 17.54263 | 157.4941 | 70.51845 | 30.16288 | 28.21591 | 41.25505 | 4.14358 | 146.3159 | 49.73433 | 58.94661 | 632.2778 |
| 1997 | 23.36469 | 4.57707 | 18.10362 | 172.9166 | 62.93452 | 33.70782 | 29.54398 | 39.36396 | 4.12633 | 164.3666 | 52.41591 | 58.57007 | 663.9911 |
| 1998 | 25.31875 | 3.7337 | 19.4008 | 169.3689 | 64.93807 | 38.22117 | 27.84789 | 39.08118 | 4.38914 | 163.7264 | 54.49104 | 59.2997 | 669.8167 |
| 1999 | 24.29292 | 4.55531 | 17.51267 | 175.2583 | 67.4695 | 38.63305 | 29.00986 | 38.26687 | 4.48299 | 172.8469 | 55.01197 | 61.05946 | 688.3997 |
| 2000 | 25.2887 | 4.26857 | 17.93146 | 172.5556 | 65.67294 | 39.75545 | 29.77793 | 36.21488 | 5.15424 | 191.7864 | 51.7158 | 63.92528 | 704.0473 |
| 2001 | 26.20386 | 6.29773 | 19.32213 | 177.7461 | 70.53834 | 39.96574 | 30.39587 | 44.3714 | 5.53236 | 195.5946 | 50.35953 | 70.86833 | 737.196 |
| 2002 | 27.70121 | 6.56812 | 19.6735 | 188.8748 | 73.2371 | 39.26101 | 34.97144 | 43.94973 | 7.10124 | 199.9743 | 51.46457 | 74.24318 | 767.0202 |
| 2003 | 27.90813 | 6.98176 | 20.18842 | 197.4258 | 67.60532 | 43.58198 | 35.70823 | 42.34238 | 7.97955 | 229.2154 | 51.06403 | 72.60925 | 802.6103 |
| 2004 | 30.04089 | 7.06758 | 21.23862 | 211.3123 | 73.43224 | 47.02407 | 39.08187 | 40.29516 | 8.60989 | 259.5141 | 52.96562 | 74.48086 | 865.0632 |
| 2005 | 31.11726 | 7.23262 | 22.25807 | 215.2574 | 78.08508 | 50.77316 | 40.71064 | 45.38736 | 8.48946 | 265.4204 | 57.28808 | 78.7649 | 900.7843 |
| 2006 | 31.49121 | 8.10085 | 24.12864 | 227.1841 | 74.3584 | 51.32774 | 37.84839 | 41.11301 | 11.55621 | 261.9042 | 69.06457 | 85.46452 | 923.5419 |
| 2007 | 33.39287 | 9.16842 | 24.10169 | 230.3485 | 83.87088 | 51.02943 | 38.33425 | 39.30205 | 12.41426 | 250.1095 | 73.50196 | 90.77959 | 936.3534 |
| 2008 | 35.96839 | 9.51201 | 24.74248 | 239.0164 | 89.92338 | 53.47209 | 39.28237 | 41.79489 | 14.49988 | 267.9018 | 77.41374 | 92.60482 | 986.1322 |
| 2009 | 40.92244 | 10.90303 | 28.40337 | 249.1096 | 98.00965 | 60.07814 | 40.72797 | 34.50472 | 16.80213 | 287.7539 | 89.02891 | 93.37972 | 1049.624 |
| 2010 | 40.37551 | 12.70115 | 30.78287 | 242.2579 | 97.629 | 61.98131 | 42.43035 | 34.86826 | 17.44703 | 301.1944 | 93.88286 | 98.99133 | 1074.542 |
| 2011 | 43.71223 | 12.79676 | 27.32671 | 284.5735 | 120.6283 | 54.80571 | 39.35421 | 34.55169 | 18.39418 | 323.8814 | 95.66641 | 104.0655 | 1159.757 |
